# Dopamine transporter binding in the brain is linked to irritable bowel syndrome in Parkinson's disease

**DOI:** 10.1002/brb3.3097

**Published:** 2023-05-30

**Authors:** Kirsi Murtomäki, Juho Joutsa, Tuomas Mertsalmi, Elina Jaakkola, Elina Mäkinen, Reeta Levo, Mikael Eklund, Simo Nuuttila, Eero Pekkonen, Tommi Noponen, Toni Ihalainen, Valtteri Kaasinen, Filip Scheperjans

**Affiliations:** ^1^ Department of Neurology Helsinki University Hospital Helsinki Finland; ^2^ Clinicum University of Helsinki Helsinki Finland; ^3^ Clinical Neurosciences University of Turku Turku Finland; ^4^ Neurocenter Turku University Hospital Turku Finland; ^5^ Turku PET Centre Turku University Hospital Turku Finland; ^6^ Turku Brain and Mind Center University of Turku Turku Finland; ^7^ Department of Clinical Physiology and Nuclear Medicine Turku University Hospital Turku Finland; ^8^ Department of Medical Physics Turku University Hospital Turku Finland; ^9^ Clinical Physiology and Nuclear Medicine University of Helsinki and Helsinki University Hospital Helsinki Finland

**Keywords:** dopamine transporter, gastrointestinal symptoms, nonmotor symptoms, Parkinson's disease, SPM

## Abstract

**Background:**

Gastrointestinal symptoms are common in Parkinson's disease (PD), but their neurophysiological correlates are not well understood. We recently reported that functional gastrointestinal symptoms were not associated with asymmetry per se but might be associated with lower left striatal dopamine transporter (DAT) binding. The purpose of this study was to further investigate if specific gastrointestinal symptoms associate with monoamine transporter changes in specific striatal or extrastriatal areas.

**Methods:**

Ninety PD patients, who underwent DAT ¹^2^ ^3^I‐FP‐CIT SPECT imaging, were assessed using the MDS‐Unified Parkinson's Disease Rating Scale part III, Rome III, and Wexner constipation score. DAT binding was calculated from striatal subregions using region‐to‐occipital cortex ratio. Voxel‐wise analysis was used to assess the relationship between gastrointestinal symptoms and striatal DAT and extrastriatal serotonin transporter (SERT) binding.

**Results:**

Irritable bowel syndrome (IBS) criteria were fulfilled in 17 patients and were linked to higher ¹^2^ ^3^I‐FP‐CIT binding in the right posterior putamen and adjacent areas as compared to patients without IBS. No other significant associations between gastrointestinal symptoms and DAT or SERT binding were found.

**Conclusions:**

These findings suggest that PD patients with IBS may have higher DAT binding in the right hemisphere. This finding implicates alterations of brain neurotransmitter physiology in the gastrointestinal symptoms of PD patients.

## Graphical abstract

Our study suggests that Parkinson's disease (PD) patients with irritable bowel syndrome may have higher striatal dopamine transporter binding in the right hemisphere. This finding implicates alterations of brain neurotransmitter physiology in the gastrointestinal symptoms of PD patients.

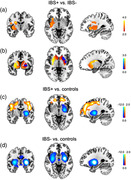

## 1 INTRODUCTION

Parkinson's disease (PD) is a very common progressive neurodegenerative disease characterized by a variety of motor and nonmotor manifestations. The cardinal motor symptoms bradykinesia and rigidity are linked to the progressive degeneration of nigral dopaminergic neurons (Pirker, 2003). These neurons project their axons to the striatum enabling imaging of presynaptic striatal dopamine transporter (DAT) binding.

Nonmotor symptoms (NMS) may precede motor symptoms by several years (Chaudhuri et al., 2005). These include REM sleep behavior disorder, orthostatic hypotension, depression, pain, fatigue, and a variety of gastrointestinal symptoms (Abbott et al., 2001; Maass & Reichmann, 2013; Pfeiffer, 2018; Poirier et al., 2016). It has been observed that a significant number of PD patients also fulfill the symptomatic criteria of irritable bowel syndrome (IBS) (Mertsalmi et al., 2017). IBS is defined as recurrent abdominal pain or discomfort—experienced at least 3 days a month in the last 3 months before filling the questionnaire (for women, the pain does not occur only during menstrual bleeding)—that is associated with two or more of the following features: (i) improvement with defecation, and onset associated with a change in (ii) frequency or (iii) form of stool (Longstreth et al., 2006). IBS is more complex than just constipation and they are mutually exclusive disorders (Mertsalmi et al., 2017).

The pathological mechanisms behind the NMS are not well understood, although autopsy studies have shown α‐synuclein pathology throughout the spinal cord and peripheral autonomic nervous system in PD patients that could play a role (Beach & Adler et al., 2010). Both genetics and environmental triggers, as well as modifiers for PD such as pesticides, toxins, and altered gut microbiota, have been linked to the pathogenesis of NMS (Chen et al., 2022). A recently introduced α‐synuclein origin site and connectome (SOC) model suggests that, in a subgroup of patients, the neuropathology initiates in the peripheral autonomic nervous system, most probably in the gut (body‐first subtype). This could lead to a more symmetrical central α‐synuclein pathology and a higher nonmotor symptom burden when compared to the brain‐first pathology (Borghammer, 2021).

DAT imaging with single‐photon emission computerized tomography (SPECT) is being used as an in vivo biomarker of nigrostriatal neuron loss in PD, and the overall striatal DAT binding correlates well with the Hoehn and Yahr stage (Pirker, 2003). On the other hand, the relation between NMS, and especially gastrointestinal symptoms (GIS), and DAT binding has been investigated in only few studies with inconclusive results (Hinkle et al., 2018; Kim & Jun, 2019; Murtomäki et al., 2022; van Deursen et al., 2020).

The connection between GIS and asymmetrical nigrostriatal dopaminergic degeneration (dopaminergic degeneration starting in one hemisphere and remaining more pronounced in that hemisphere) is poorly understood. van Deursen et al. (2020) found that autonomic symptoms, mainly gastrointestinal (GI) and cardiovascular symptoms, were related to lower DAT binding ratios in the right caudate nucleus. In a recent study, we investigated the connection between the degree of asymmetry of striatal dopaminergic defect and GIS in PD (Murtomäki et al., 2022). While we found no significant differences in GIS between symmetric and asymmetric cases, lower left‐ versus right‐sided putaminal DAT uptake, as measured using asymmetry indices (mean putamen asymmetry index calculated as AI = (putamen_highest_ – putamen_lowest_)/(putamen_highest_ + putamen_lowest_)), was associated with increased reported GIS and constipation. Multiple studies investigated the association of IBS and PD, and in a systematic review and meta‐analysis by Lu et al. (2022), the overall risk for PD in IBS patients was significantly higher as compared to the general population (odds ratio: 1.5). Therefore, intestinal dysfunction may be a prodromal PD symptom (Lu et al., 2022).

In addition to high affinity to the presynaptic DAT, the ¹^2^ ^3^I‐FP‐CIT ligand has a modest affinity for the presynaptic serotonin transporter (SERT) (Abi‐Dargham et al., 1996; Joling et al., 2019). Therefore, it is possible to simultaneously use ¹^2^ ^3^I‐FP‐CIT as a proxy for the integrity of both the striatal dopaminergic and the extrastriatal serotonergic system in vivo (Ziebell et al., 2010).

In this analysis, we aimed to get further insight into the relationship between asymmetric DAT binding and GI symptoms and extended the analysis to extrastriatal regions. To this end, we here investigated striatal and extrastriatal ¹^2^ ^3^I‐FP‐CIT binding in relation to GIS in the same PD population using voxel‐wise analysis (Murtomäki et al., 2022).

## 2 METHODS

This cross‐sectional clinical and imaging study consisted of patients imaged with ^123^I‐FP‐CIT SPECT because of parkinsonism or tremor referred to imaging by their neurologist (Murtomäki et al., 2022). A total of 401 patients were recruited into the study between the years 2015 and 2019 at Helsinki and Turku University Hospitals, when they came to the imaging visit (NMDAT study; ClinicalTrials.gov identifier NCT02650843). The study subjects were required to be aged 18 or over and to be able to understand and answer the questionnaires in Finnish. Subjects with any limitation affecting their ability to understand the informed consent, such as significant mental health problems or cognitive problems (Mini–Mental State Examination [MMSE] <18), were excluded.

The study subjects were interviewed and clinically examined while they were waiting for the imaging. The imaging was performed for 399 patients. Of the imaged patients, 248 returned the questionnaires regarding GIS. Out of these patients, 90 patients were eventually diagnosed with PD and were investigated in the present study. The diagnosis of PD was made according to the UK Parkinson's Disease Society Brain Bank clinical diagnostic criteria and required furthermore an abnormal ^123^I‐FP‐CIT SPECT imaging result (Gibb & Lees, 1988).

The study was approved by the Ethics Committee of the Turku University Hospital and was conducted according to the principles of the Declaration of Helsinki. Written informed consent was obtained from all participants in the study.

### 2.1 Clinical features

All patients were clinically examined before imaging. The clinical examinations included an interview, MDS‐Unified Parkinson's Disease Rating Scale part III (MDS‐UPDRS part III) (Goetz et al., 2008) in ON state, and MMSE (Folstein et al., 1975). All investigators had completed the MDS‐UPDRS Training Program and Exercise. For the parkinsonian motor features, the MDS‐UPDRS part III total score was calculated. A permission to use MDS‐UPDRS part III was asked and granted by Movement Disorder Society.

GIS were evaluated with Wexner constipation score (Agachan et al., 1996) and Rome III questionnaire (Drossman & Dumitrascu, 2006). If there were more than 20% of answers missing, the value was labeled as missing. If there were less than 20% of answers missing, the values were corrected for the missing ones (value × questions/(questions – unanswered questions)) (Joutsa et al., 2012) for Rome III questionnaire and Wexner score as in our previous article (Murtomäki et al., 2022).

We used Rome III criteria for the identification of functional gastrointestinal disorders (FGIDs): IBS and it's subcategories, and functional dyspepsia.

Based on the significant results from our previous article (Murtomäki et al., 2022), we decided to use FGIDs, IBS, IBS constipation, functional dyspepsia, and Wexner scores in our analyses.

### 2.2 SPECT imaging and data analysis

SPECT imaging was carried out with different systems (and collimators) in different sites (Siemens Symbia T6 [low‐energy high‐resolution, LEHR], Phillips Brightview XCT [LEHR], two GE Infinia II Hawkeyes [LEHRs], Siemens Intevo [Fanbeam], GE NM/CT 670 ES [LEHR]) and the results were scanner‐specific corrected and patient‐age corrected for the specific binding ratio (SBR) (Albert et al., 2016). Specific ¹^2^ ^3^I‐FP‐CIT binding was calculated from striatal subregions (caudate, anterior putamen, posterior putamen) using region‐to‐occipital cortex ratio, as in our earlier studies (Kaasinen et al., 2014). The image quality was inspected visually by the investigators. To mitigate the potential confounding effects of different scanners, the SPECT device was calibrated using instructions for camera corrections from the ENC‐DAT database (Varrone et al., 2013).

The classification of patients to normal and abnormal ¹^2^ ^3^I‐FP‐CIT binding groups was based on the automated semiquantitative BRASS‐analysis (Hermes Medical Solutions AB, Stockholm, Sweden). The limit for abnormal area was more than 2 standard deviations below the reference mean in any of the six analyzed regions. Images with borderline results were re‐evaluated by a movement disorder specialist with extensive experience in brain dopamine imaging (V.K.).

### 2.3 Statistical analyses

IBM SPSS statistics Version 25 was used in all of the analyses. Normality was evaluated visually from histograms together with Shapiro–Wilk test for continuous variables. Independent sample *t*‐test, Mann–Whitney *U* test, and Chi‐square test were used to investigate group differences in the continuous and categorical variables. Assumptions for regression analyses were checked and met. Linear regression was used with the anatomical regions of interest (ROIs; right and left posterior putamen, right and left anterior putamen, right and left caudate nucleus) SBR as the dependent variable, and sex, age, UPDRS part III total score, and IBS as the independent variable. Same calculations were also done with Wexner score as the independent variable. Statistical significance was defined as *p* < .05. We performed Bonferroni correction accounting for all GI symptoms analyzed and the results remained significant (*p*_adj_ < .05).

General linear model implemented in Statistical Parametric Mapping software version 12 (SPM12, https://www.fil.ion.ucl.ac.uk/spm/software/spm12/) was used to investigate the relationship of ¹^2^ ^3^I‐FP‐CIT binding between DAT and SERT, and each GIS voxel‐by‐voxel with age, sex, and UPDRS part III total score as covariates. These covariates were chosen because they have been shown to be associated with ¹^2^ ^3^I‐FP‐CIT binding (Honkanen et al., 2021; Pirker, 2003). The ratio images were smoothed using 8 mm at full‐width–half‐maximum Gaussian kernel to improve the signal‐to‐noise ratio. An analysis mask including the frontal lobes, cingulate cortex, basal ganglia, thalamus, media temporal lobe, and midbrain was created using WFU pickatlas (version 3.05) (Eklund et al., 2022). Cluster‐level family‐wise error (FWE) correction was used to test for significance (with *p* < .005 uncorrected and after .05 FWE cluster‐level corrected *p*‐values). For GIS showing significant association with ¹^2^ ^3^I‐FP‐CIT binding in the striatum, we created unthresholded striatal maps to further visualize the exact regions within the striatum using unthresholded *t*‐maps.

## 3 RESULTS

### 3.1 GIS in PD patients

Basic cohort characteristics are summarized in Tables 1 and 2. A total of 90 PD patients were analyzed, and 32 (35.6%) of these patients fulfilled the Rome III criteria for at least one FGID. Seventeen (18.9%) patients fulfilled the criteria for IBS, of which five patients fulfilled criteria for constipation‐predominant IBS subtype. For the other FGIDs, functional dyspepsia was the most prevalent (*n* = 14, 15.5%). Wexner total scores were 5.04 ± 4.05 and Rome III constipation subscore was 5.91 ± 4.77. Twenty‐six (28.9%) patients were using at least some dopaminergic medication.

**TABLE 1:** Basic characteristics of PD patients.

Characteristics	PD
Number of patients	90
Age, mean ± SD	65.29 ± 9.97
Sex; Male, *n* (%)	46 (51.1)
MMSE, mean ± SD	27.51 ± 1.86
Motor symptoms in months, mean ± SD	28.92 ± 31.09
Hoehn and Yahr score, mean ± SD	1.99 ± 0.71
H&Y 1, *N* (%)	21 (23.3)
H&Y 2, *N* (%)	51 (56.7)
H&Y 3, *N* (%)	16 (17.8)
H&Y 4, *N* (%)	2 (2.2)
MDS‐UPDRS III total score (ON), mean ± SD	34.72 ± 13.57
Functional gastrointestinal disorders, *N* (%)	32 (35.6)
IBS, *n* (%)	17 (18.9)
IBS‐C, *n* (%)	5 (5.6)
Functional dyspepsia, *n* (%)	14 (15.5)
Functional constipation, *n* (%)	4 (4.4)
Functional bloating, *n* (%)	3 (3.3)
Functional diarrhea, *n* (%)	1 (1.1)
Wexner total score, mean ± SD	5.04 ± 4.05
Rome III constipation items, mean ± SD	5.91 ± 4.77
BAI score, mean ± SD	10.36 ± 6.72
BDI score, mean ± SD	6.87 ± 6.05
Patients with dopaminergic medication, N(%)	26 (28.9)
Mean levodopa equivalent daily dose, mean ± SD (range)	82.27 ± 155.36 (0–600)
Right posterior putamen SBR, mean ± SD	1.08 ± 0.55
Left posterior putamen SBR, mean ± SD	0.96 ± 0.40
Right anterior putamen SBR, mean ± SD	1.68 ± 0.65
Left anterior putamen SBR, mean ± SD	1.63 ± 0.54
Right nucleus caudatus SBR, mean ± SD	2.14 ± 0.64
Left nucleus caudatus SBR, mean ± SD	2.19 ± 0.65

*Note*: Wexner is a constipation questionnaire. Rome III is assessing functional gastrointestinal disorders.

Abbreviations: BAI, Beck Anxiety Inventory; BDI, Beck Depression Inventory; IBS, irritable bowel syndrome; IBS‐C, irritable bowel syndrome constipation subtype; MDS‐UPDRS III, MDS‐Unified Parkinson's Disease Rating Scale part III; MMSE, Mini–Mental State Examination; *N*, number of patients; SBR, specific binding ratio; SD, standard deviation.

**TABLE 2:** PD patients divided into groups with IBS and without IBS.

Characteristics	IBS+	IBS–	*p* value
Number of patients	17	73	
Age, mean ± SD	66.53 ± 7.59	65.00 ± 10.47	.83
Sex; Male, N (%)	6 (35.3)	40 (54.8)	.15
MMSE, mean ± SD	27.18 ± 1.85	27.59 ± 1.87	.40
Motor symptoms in months, mean ± SD	25.71 ± 17.23	29.67 ± 33.56	.80
Hoehn and Yahr score, mean ± SD	1.82 ± 0.73	2.03 ± 0.71	.30
MDS‐UPDRS III total score (ON), mean ± SD	30.24 ± 9.17	35.77 ± 14.25	.13
Wexner total score, mean ± SD (N = 88)	9.88 ± 3.76	3.88 ± 3.18	<.001
Rome III constipation items, mean ± SD	10.58 ± 4.86	4.87 ± 4.11	<.001
BAI score, mean ± SD	14.00 ± 5.76	9.43 ± 6.67	.03
BDI score, mean ± SD	7.59 ± 5.26	6.70 ± 6.25	.37
Patients with dopaminergic medication, *N* (%)	5 (29.4)	21 (28.8)	.89
Mean levodopa equivalent daily dose, mean ± SD (range)	38.35 ± 69.69 (0–200)	92.64 ± 168.08 (0–600)	.68
Right posterior putamen SBR, mean ± SD	1.29 ± 0.93	1.03 ± 0.40	.47
Left posterior putamen SBR, mean ± SD	0.91 ± 0.37	0.97 ± 0.41	.77
Right anterior putamen SBR, mean ± SD	1.90 ± 0.91	1.63 ± 0.56	.40
Left anterior putamen SBR, mean ± SD	1.63 ± 0.65	1.63 ± 0.51	.70
Right caudate SBR, mean ± SD	2.37 ± 0.88	2.09 ± 0.57	.32
Left caudate SBR, mean ± SD	2.24 ± 0.84	2.18 ± 0.61	.98
Right mean putamen, mean ± SD	1.60 ± 0.90	1.33 ± 0.46	.45
Left mean putamen, mean ± SD	1.27 ± 0.47	1.30 ± 0.43	.75

*Note*: Wexner is a constipation questionnaire. Rome III is assessing functional gastrointestinal disorders.

Abbreviations: BAI, Beck Anxiety Inventory; BDI, Beck Depression Inventory; IBS, irritable bowel syndrome; MDS‐UPDRS III, MDS‐Unified Parkinson's Disease Rating Scale part III; MMSE, Mini–Mental State Examination; *N*, number of patients; SBR, specific binding ratio; SD, standard deviation.

The PD patients with IBS and without IBS (Table 2) did not significantly differ in terms of age, sex, duration of motor symptoms, Hoehn and Yahr stage, MDS‐UPDRS part III total scores, or MMSE scores. As expected, patients with IBS had higher Wexner constipation scores (*p* < .001) and higher Rome III constipation subscores (*p* < .001). Five patients with IBS (29.4%) and 21 without IBS (28.8%) were using dopaminergic medication. There were no significant differences in the mean levodopa equivalent daily dose between the groups.

### 3.2 SPECT imaging and GIS

**FIGURE 1:**
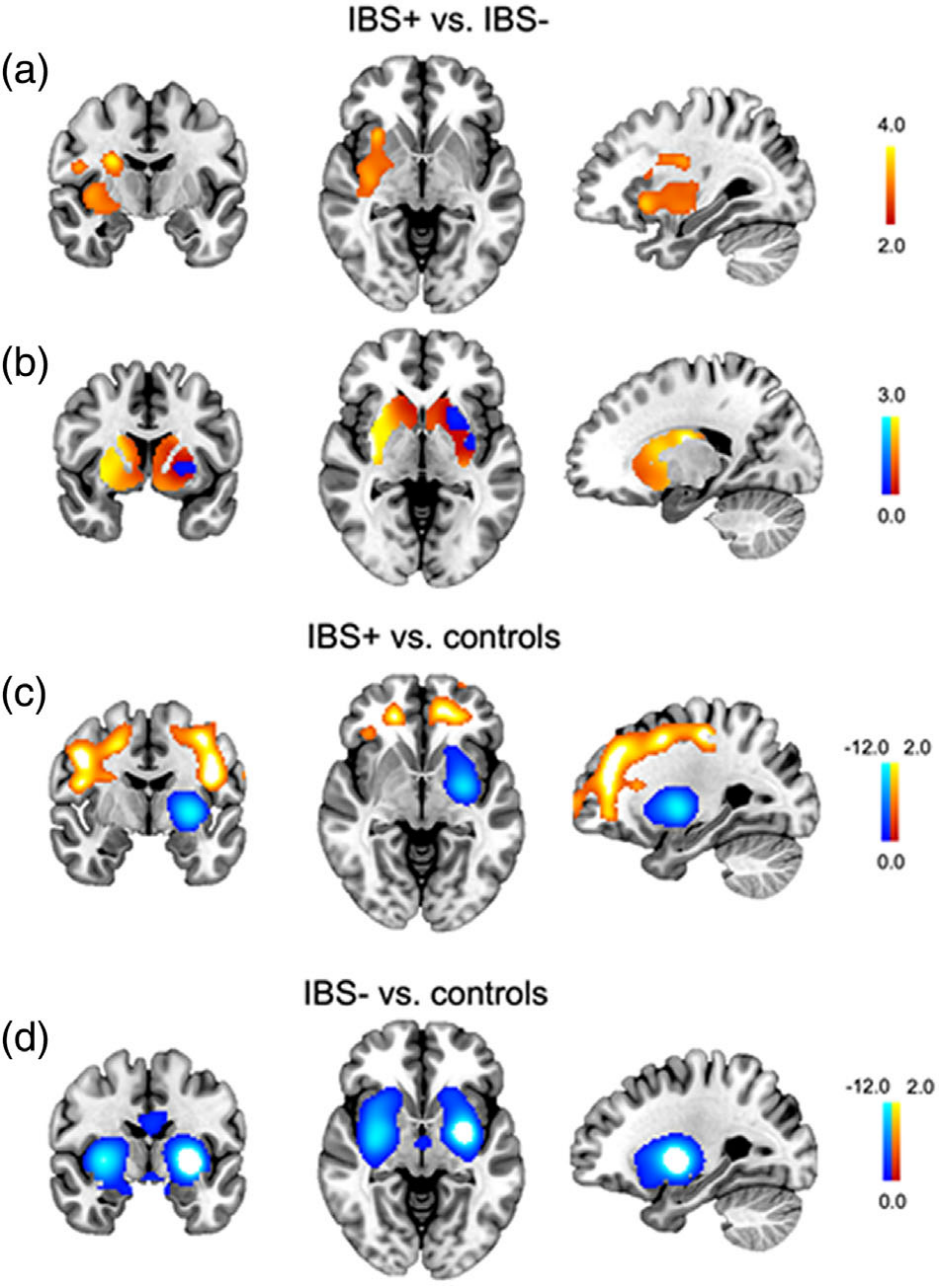
A. Brain regions showing significantly higher ^123^I‐FP‐CIT binding in patients with IBS compared to patients without IBS (cluster size 2327 voxels, peak at MNI‐coordinate 24 −10 22, P_uncorr_<0.005, PFWE=0.008). B. Unthresholded striatal t map of the contrast patients with IBS > patients without IBS demonstrating higher binding in the right sensorimotor striatum. Note that panel B is intended to visualize the group differences in the whole striatum and not for statistical testing of any hypotheses. C‐D. PD patients with IBS (C) and without IBS (D) compared to healthy controls to illustrate the overall ^123^I‐FP‐CIT binding abnormality in these patients (PFWE<0.05 for all clusters). Left side in the figure = right side of the brain.

When comparing ¹^2^ ^3^I‐FP‐CIT binding with voxel‐based SPM between IBS+ and IBS– patients, we found a cluster of higher binding in IBS patients mostly in the right posterior putamen, extending to the globus pallidus, internal capsule, claustrum, insula, and adjacent white matter (Figure 1). In addition, Wexner constipation scores were significantly associated with higher ¹^2^ ^3^I‐FP‐CIT binding in a widespread area bilaterally in the dorsal frontal cortex (cluster size: 15,637 voxels, peak at Montreal Neurological Institute coordinates: −6, −22, 74, *p*_FWE_ < .001); however, the average binding in this cluster did not exceed the uptake in the reference regions, indicating this finding was not driven by true differences in specific monoamine transporter binding. There were no significant associations for other symptom scores.

We also analyzed the relationship between IBS and SBRs using ROIs in the right and left posterior putamen, right and left anterior putamen, and right and left caudate nucleus. There were no clinically significant associations in any of the ROIs (Table 2). In the linear regression model, there was a trend toward higher binding in the right posterior putamen in patients with IBS, supporting the results of the voxel‐wise analysis (i.e., analyses covering each of the voxels in the search volume, as opposed to ROI analyses that average the signal across all voxels in the ROI) (Table 3). The results were in line with SPM results, but not significant (*p* = .089). For absolute SBR values, please see Table S1. In line with the imaging findings, IBS+ patients more frequently had right‐predominant motor symptoms (78%) as compared to IBS– patients (42%) (*p* = .058).

**TABLE 3:** All PD patients (*N* = 90), linear regression analyses with IBS for each ROI.

ROI	Variable	*β*	*p* value	Adjusted *R* ^2^
Right posterior putamen	IBS	.186	.089	
	Age	−.138	.26	
	Sex	−.011	.92	
	UPDRS part III total score	−.060	.65	.023
Left posterior putamen	IBS	−.063	.56	
	Age	−.190	.12	
	Sex	.014	.90	
	UPDRS part III total score	−.140	.28	.039
Right anterior putamen	IBS	.143	.17	
	Age	−.249	.034	
	Sex	−.082	.45	
	UPDRS part III total score	−.129	.30	.113
Left anterior putamen	IBS	−.006	.17	
	Age	−.354	.034	
	Sex	−.083	.45	
	UPDRS part III total score	−.081	.30	.136
Left nucleus caudatus	IBS	.008	.93	
	Age	−.374	.001	
	Sex	−.158	.11	
	UPDRS part III total score	−.190	.093	.267
Right nucleus caudatus	IBS	.152	.13	
	Age	−.306	.007	
	Sex	−.093	.37	
	UPDRS part III total score	−.169	.16	.190

Abbreviation: adjusted *R* ^2^, proportion of variance explained by the regression model; IBS, irritable bowel syndrome; *N*, number of patients; ROI, region of interest; *β*, standardized regression coefficient.

## 4 DISCUSSION

This study was prompted by our previous finding that lower left‐ versus right‐sided putaminal DAT binding, based on asymmetry indices, was associated with a higher prevalence of FGIDs in PD patients (Murtomäki et al., 2022). Our current more extended voxel‐based analysis on FGIDs suggests that in fact IBS is associated with higher DAT binding specifically in the right hemisphere covering striatal and extrastriatal regions, including the putamen, extending to the globus pallidus, internal capsule, claustrum, insula, and adjacent white matter, instead of left‐predominant reduction of DAT binding (which would both result in comparable asymmetry indices). This finding gives more insight concerning the possible etiology of IBS symptoms in PD. For the other GIS, there were no clinically significant associations with DAT or SERT binding.

Voxel‐wise analysis has been previously used with both SPECT and positron emission tomography (PET) imaging with various ligands to study PD patients. Recently, Nicastro et al. (2020) used voxel‐wise analysis to assess correlations of motor and nonmotor symptoms with striatal and extrastriatal ¹^2^ ^3^I‐FP‐CIT binding in the Parkinson's Progressive Markers Initiative cohort. They found a decreased binding in the striatum and extrastriatal regions including the pallidum, amygdala, and insula in early PD patients compared to control subjects, confirming findings from previous studies (Lee et al., 2018; Pilotto et al., 2019). There was a trend association between higher geriatric depression scores and lower pallidal DAT binding. Significant associations between autonomic scale GI subscore (SCOPA‐AUT) and lower uptake in mean putamen and caudate nucleus were also found. This was in line with the results from a previous study by Hinkle et al. (2018). Also, SCOPA‐AUT urinary subscore was negatively correlated with striatal uptake as in a previous study by Kim and Jun (2019). However, they did not report any lateralization or higher binding in contrast to our results.

Colloby et al. (2004) used ^123^I‐FP‐CIT SPECT imaging with voxel‐wise analysis to investigate differences in striatal binding in subjects with dementia with Lewy bodies (DLB), Alzheimer's disease (AD), and PD and healthy age‐matched controls. They found a significant bilateral reduced uptake in the caudate nucleus and posterior and anterior putamen both in PD patients and subjects with DLB compared to AD subjects and controls supporting the use of voxel‐wise analysis in PD.

In a ^18^F‐Dopa PET imaging study, Ito and colleagues showed reductions in both striatal and nigral brain dopaminergic functions in early PD (Ito et al., 1999). In another study with ^18^F‐FP‐CIT PET, there was significantly decreased ^18^F‐FP‐CIT binding in the contralateral putamen in early‐stage PD patients compared to controls (Ma et al., 2002). To our knowledge, PET imaging with voxel‐wise analyses has not been used to study GIS and PD.

Previously, the lateralization of dopaminergic degeneration and GIS in PD has been studied in only few studies. Deursen and colleagues observed that autonomic symptoms, mainly cardiovascular and SCOPA‐AUT GI subscores, were associated with lower ¹^2^ ^3^I‐FP‐CIT binding ratios in the right caudate nucleus (van Deursen et al., 2020). A negative association was shown between DAT binding and SCOPA‐AUT GI subscores in the right caudate nucleus. This is in contrast to our result of higher binding in the right posterior putamen linked to IBS. In Deursen's study, the seven‐question SCOPA‐AUT GI subdomain, with questions on swallowing, drooling, and early fullness, was used and only three questions focused on bowel symptoms (Visser et al., 2004), whereas we used the Rome III questionnaire to evaluate different FGIDs. The use of these different questionnaires might explain some of the differences between the results. In a Swedish study by Liu et al., IBS diagnosis was associated with a 44% higher risk of PD (Liu et al., 2021). In another article by Mertsalmi et al., the association between IBS and PD was confirmed but may have been explained by reverse causation and detection bias (Mertsalmi et al., 2021). Mishima et al. found the prevalence of IBS in PD to be 17.0%, which is a higher prevalence compared with the general population (Mertsalmi et al., 2017; Mishima et al., 2017). Also, in a study by Lai et al. (2014), the adjusted hazard ratio of PD associated with IBS was 1.48, compared with the non‐IBS group. So, IBS seems to be more common in PD patients. The high prevalence of IBS symptoms in PD and the suggestion that IBS may be a risk factor for PD warrant further investigation of the underlying mechanisms (Mertsalmi et al., 2017; Zhang et al., 2021).

It is unclear whether the increased DAT signal in the right posterior putamen reflects a lesser degree of neurodegeneration or upregulation of DAT in PD patients reporting IBS symptoms. To our knowledge, no previous studies have used DAT imaging in IBS patients. In MRI studies of IBS patients, the most consistent structural and functional alterations are found in the somatosensory network, including the globus pallidus, putamen, and caudate nucleus (Hillestad et al., 2022). A diffusion tensor imaging (DTI) study on IBS patients found white matter damage in the splenium of the corpus callosum, the right retrolenticular area of the internal capsule, and the right superior corona radiata (Fang et al., 2017). In another DTI study, decreased fractional anisotropy in the right dorsal cingulum bundle in adolescent female patients with IBS was found when compared to controls (Hubbard et al., 2018). IBS patients with alexithymia showed also altered brain responses to rectal distension in the right insula (Kano et al., 2020). Further, increased SERT binding in the midbrain and thalamus has been associated with functional dyspepsia (Tominaga et al., 2015). These results suggest that IBS is associated with central nervous system changes, and the basal ganglia and lateralization might have an effect on IBS and other FGIDs. On the other hand, elevated SERT has been also found in PD patients with depression and it was speculated to be caused by upregulation, so upregulation might be one explanation (Boileau et al., 2008). Our finding has not been reported before in studies on PD and NMS and warrants more investigation on that topic.

A strength of our study is that the diagnosis of PD was based on ¹^2^ ^3^I‐FP‐CIT SPECT and the opinion of a movement disorder specialist. The patients were being followed up to 4 years to diminish the possibility of false PD diagnosis, and the mean follow‐up time at the time of our analyses was 30 months (2–57 months). The patients were sent to imaging for diagnostic purposes and most of them had early‐stage symptoms without long use of dopaminergic medication. On the other hand, selection bias cannot be excluded, because patients were referred to ¹^2^ ^3^I‐FP‐CIT SPECT imaging, which may reflect some initial diagnostic uncertainty.

While we used multiple GIS questionnaires, the lack of objective measurements of GI functions is a slight limitation. However, it has been shown that PD patients and non‐PD patients with functional constipation have severe functional dysmotility of the colon and rectum based on objective GI investigations (Zhang et al., 2021), supporting the use of the Rome questionnaire.

It should also be noted that, unlike the voxel‐wise analyses, our ROI analyses failed to identify significant associations between IBS and ¹^2^ ^3^I‐FP‐CIT binding. However, there was a trend‐level finding in the right posterior putamen, supporting our voxel‐wise findings. The ROI analysis averages voxels from an anatomical region with the assumption that the entire anatomical region has the same function, which often is not a valid assumption. In fact, the cluster associated with IBS covered only the ventral part of the posterior putamen extending to other anatomical regions, suggesting that there may be specific subregions in the putamen and other brain regions driving this effect. It should be noted that since SPECT generally is less sensitive and has poorer spatial resolution compared to PET, investigating IBS in PD using PET imaging could possibly be useful to further improve the spatial specificity of our findings.

Patient‐reported GIS reflect the patients’ subjective experience of symptoms, which is clinically most relevant and likely results from a combination of underlying mechanisms. The etiologies behind the GIS are very complex, and altered bacterial flora (Mertsalmi et al., 2017), small intestinal bacterial overgrowth, increased intestinal permeability as well as abnormal gut motility might be etiologic factors (Mulak & Bonaz, 2015). Furthermore, there is evidence for connections between mental health and gut health. A study on anxiety, depression, and GIS in PD suggested a possible cyclical relationship, where GIS increase an individual's risk for worsening mood symptoms, which in turn increase their risk for worsening GIS (Jones et al., 2021). Taken together, the patient's symptom experience is a combination of multiple mechanisms.

PD patients tend to have a larger number of different NMS compared with normal controls, and NMS tend to be more frequent and more severe (Kim et al., 2013; Krishnan et al., 2011). The NMS have a large effect on the quality of life for the patient and caregiver and also on institutionalization rates (Chaudhuri et al., 2006; Pfeiffer, 2016). It is very important to investigate the NMS’ etiologies to develop novel therapeutic strategies. Our study suggests a link between the lateralization of DAT availability and GIS in PD and elucidates the role of the dopaminergic system in the gut–brain axis in PD patients.

## AUTHOR CONTRIBUTIONS

**Kirsi Murtomäki**: Data curation (lead); formal analysis (lead); funding acquisition (equal); investigation (equal); visualization (equal); writing—original draft preparation (lead); writing—review and editing (equal). **Juho Joutsa**: Conceptualization (equal); formal analysis (equal); methodology (supporting); project administration (supporting); supervision (supporting); writing—original draft preparation (supporting); writing—review and editing (equal). **Tuomas Mertsalmi**: Data curation (supporting); investigation (supporting); methodology (supporting); writing—review and editing (supporting). **Elina Jaakkola**: Data curation (supporting); formal analysis (supporting); investigation (equal); project administration (supporting); writing—review and editing (supporting). **Elina Mäkinen**: Data curation (supporting); formal analysis (supporting); investigation (equal); project administration (supporting); writing—review and editing (supporting). **Reeta Levo**: Data curation (supporting); investigation (equal); project administration (supporting); writing—review and editing (supporting). **Mikael Eklund**: Data curation (supporting); investigation (equal); writing—review and editing (supporting). **Simo Nuuttila**: Data curation (equal); investigation (equal); writing—review and editing (equal). **Eero Pekkonen**: Supervision (equal); writing—review and editing (supporting). **Tommi Noponen**: Data curation (supporting); investigation (supporting); project administration (supporting); resources (supporting); writing—review and editing (supporting). **Toni Ihalainen**: Investigation (supporting); formal analysis (equal); writing—review and editing (supporting). **Valtteri Kaasinen**: Funding acquisition (equal); methodology (equal); project administration (supporting); resources (equal); supervision (equal); writing—review and editing (equal). **Filip Scheperjans**: Conceptualization (lead); data curation (supporting); formal analysis (supporting); funding acquisition (lead); investigation (supporting); methodology (supporting); project administration (supporting); supervision (equal); validation (equal); writing—review and editing (lead).

## CONFLICT OF INTEREST STATEMENT

K.M.: Grants from the Hospital District of Helsinki and Uusimaa and the Maire Taponen Foundation. J.J.: Lecturer honoraria from Lundbeck; research grants from the Finnish Medical Foundation, the Sigrid Juselius Foundation, the Instrumentarium Research Foundation, the Finnish Foundation for Alcohol Studies, the University of Turku, private donation, and the Turku University Hospital (Finnish governmental research funding); and conference travel support from Abbvie. T.M.: Lecture fee from Merck. E.J.: Grant from the Finnish Medical Foundation. E.M.: Grants from the Finnish Neurological Society, the Maire Taponen Foundation, and the Sigrid Juselius Foundation. E.P.: Member of the MDS Non‐Motor Parkinson's Disease Study Group, PI in Finland: International Adroit study (Abbott DBS Registry of Outcomes for Indications over Time); Member of the advisory board: Abbvie and NordicInfu Care; Consulting fees: NordicInfu Care and Abbvie; Lecture fees: Abbott, Abbvie, and NordicInfu Care. V.K.: Member of the advisory board: Abbvie and Nordic Infucare; Consultancies: Nordic Infucare; Employment: University of Turku, Turku University Hospital, and Medishare Oy; Grants: the Turku University Foundation and the Turku University Hospital; Honoraria: Abbvie and Nordic Infucare. F.S. Grants from the Academy of Finland, the Hospital District of Helsinki and Uusimaa, the OLVI‐Foundation, the Konung Gustaf V:s och Drottning Victorias Frimurarestiftelse, the Wilhelm and Else Stockmann Foundation, the Emil Aaltonen Foundation, the Yrjö Jahnsson Foundation, the Sigrid Jusélius Foundation, and Renishaw; Honoraria: AbbVie, Axial Biotherapeutics, Orion, GE Healthcare, Merck, Teva, Bristol Myers Squibb, Sanofi, Biocodex, Lundbeck, and Biogen; Founder and CEO of NeuroInnovation Oy and NeuroBiome Ltd.; Member of the advisory board: Axial Biotherapeutics and MRM Health; Stock options: Axial Biotherapeutics.

### PEER REVIEW

The peer review history for this article is available at https://publons.com/publon/10.1002/brb3.3097.

## Supporting information


**Supplementary Table S1** SBR values and Z‐scores in PD patients in putamen and nucleus caudatus.

## Data Availability

Data available on request due to privacy/ethical restrictions.

## References

[brb33097-bib-0001] Abbott, R. D. , Petrovitch, H. , White, L. R. , Masaki, K. H. , Tanner, C. M. , Curb, J. D. , Grandinetti, A. , Blanchette, P. L. , Popper, J. S. , & Ross, G. W. (2001). Frequency of bowel movements and the future risk of Parkinson's disease. Neurology, 57(3), 456–462. 10.1212/WNL.57.3.456 11502913

[brb33097-bib-0002] Abi‐Dargham, A. , Gandelman, M. S. , DeErausquin, G. A. , Zea‐Ponce, Y. , Zoghbi, S. S. , Baldwin, R. M. , Laruelle, M. , Charney, D. S. , Hoffer, P. B. , Neumeyer, J. L. , & Innis, R. B. (1996). SPECT imaging of dopamine transporters in human brain with iodine‐123‐fluoroalkyl analogs of beta‐CIT. Journal of Nuclear Medicine, 37(7), 1129–1133. 8965183

[brb33097-bib-0003] Agachan, F. , Chen, T. , Pfeifer, J. , Reissman, P. , & Wexner, S. D. (1996). A constipation scoring system to simplify evaluation and management of constipated patients. Diseases of the Colon and Rectum, 39(6), 681–685. 10.1007/BF02056950 8646957

[brb33097-bib-0004] Albert, N. L. , Unterrainer, M. , Diemling, M. , Xiong, G. , Bartenstein, P. , Koch, W. , Varrone, A. , Dickson, J. C. , Tossici‐Bolt, L. , Sera, T. , Asenbaum, S. , Booij, J. , Kapucu, L. Ö. A. , Kluge, A. , Ziebell, M. , Darcourt, J. , Nobili, F. , Pagani, M. , Sabri, O. , … La Fougère, C. (2016). Implementation of the European multicentre database of healthy controls for [123I]FP‐CIT SPECT increases diagnostic accuracy in patients with clinically uncertain parkinsonian syndromes. European Journal of Nuclear Medicine and Molecular Imaging, 43(7), 1315–1322. 10.1007/s00259-015-3304-2 26780619

[brb33097-bib-0005] Beach, T. G. , Adler, C. H. , Sue, L. I. , Vedders, L. , Lue, L. , White, C. L., III , Akiyama, H. , Caviness, J. N. , Shill, H. A. , Sabbagh, M. N. , & Walker, D. G. (2010). Multi‐organ distribution of phosphorylated α‐synuclein histopathology in subjects with Lewy body disorders. Acta Neuropathologica, 119(6), 689–702. 10.1007/s00401-010-0664-3 20306269 PMC2866090

[brb33097-bib-0006] Boileau, I. , Warsh, J. J. , Guttman, M. , Saint‐Cyr, J. A. , Mccluskey, T. , Rusjan, P. , Houle, S. , Wilson, A. A. , Meyer, J. H. , & Kish, S. J. (2008). Elevated serotonin transporter binding in depressed patients with Parkinson's disease: A preliminary PET study with [ ^11^ C]DASB. Movement Disorders, 23(12), 1776–1780. 10.1002/mds.22212 18661545

[brb33097-bib-0007] Borghammer, P. (2021). The α‐synuclein origin and connectome model (SOC Model) of Parkinson's disease: Explaining motor asymmetry, non‐motor phenotypes, and cognitive decline. Journal of Parkinson's Disease, 11(2), 455–474. 10.3233/JPD-202481 PMC8150555 33682732

[brb33097-bib-0008] Chaudhuri, K. R. , Healy, D. G. , & Schapira, A. H. (2006). Non‐motor symptoms of Parkinson's disease: Diagnosis and management. Lancet Neurology, 5(3), 235–245. 10.1016/S1474-4422(06)70373-8 16488379

[brb33097-bib-0009] Chaudhuri, K. R. , Yates, L. , & Martinez‐Martin, P. (2005). The non‐motor symptom complex of Parkinson's disease: A comprehensive assessment is essential. Current Neurology and Neuroscience Reports, 5(4), 275–283. 10.1007/s11910-005-0072-6 15987611

[brb33097-bib-0010] Chen, H. , Wang, K. , Scheperjans, F. , & Killinger, B. (2022). Environmental triggers of Parkinson's disease ‐ Implications of the Braak and dual‐hit hypotheses. Neurobiology of Disease, 163, 105601. 10.1016/j.nbd.2021.105601 34954321 PMC9525101

[brb33097-bib-0011] Colloby, S. J. , O'brien, J. T. , Fenwick, J. D. , Firbank, M. J. , Burn, D. J. , Mckeith, I. G. , & Williams, E. D (2004). The application of statistical parametric mapping to 123I‐FP‐CIT SPECT in dementia with Lewy bodies, Alzheimer's disease and Parkinson's disease. Neuroimage, 23(3), 956–966. 10.1016/j.neuroimage.2004.06.045 15528096

[brb33097-bib-0012] Drossman, D. A. , & Dumitrascu, D. L. (2006). Rome III: New standard for functional gastrointestinal disorders. Journal of Gastrointestinal and Liver Diseases, 15(3), 237–241. 17013448

[brb33097-bib-0013] Eklund, M. , Nuuttila, S. , Joutsa, J. , Jaakkola, E. , Mäkinen, E. , Honkanen, E. A. , Lindholm, K. , Vahlberg, T. , Noponen, T. , Ihalainen, T. , Murtomäki, K. , Nojonen, T. , Levo, R. , Mertsalmi, T. , Scheperjans, F. , & Kaasinen, V. (2022). Diagnostic value of micrographia in Parkinson's disease: A study with [123I]FP‐CIT SPECT. Journal of Neural Transmission, 129, 895–904. 10.1007/s00702-022-02517-1 35624405 PMC9217822

[brb33097-bib-0014] Fang, J. , Li, S. , Li, M. , Chan, Q. , Ma, X. , Su, H. , Wang, T. , Zhan, W. , Yan, J. , Xu, M. , Zhang, Y. , Zeng, L. , Tian, J. , & Jiang, G. (2017). Altered white matter microstructure identified with tract‐based spatial statistics in irritable bowel syndrome: A diffusion tensor imaging study. Brain Imaging and Behavior, 11(4), 1110–1116. 10.1007/s11682-016-9573-y 27627891

[brb33097-bib-0015] Folstein, M. F. , Folstein, S. E. , & Mchugh, P. R. (1975). “Mini‐mental state”. A practical method for grading the cognitive state of patients for the clinician. Journal of Psychiatric Research, 12(3), 189–198. 10.1016/0022-3956(75)90026-6 1202204

[brb33097-bib-0016] Gibb, W. R. , & Lees, A. J. (1988). The relevance of the Lewy body to the pathogenesis of idiopathic Parkinson's disease. Journal of Neurology, Neurosurgery, and Psychiatry, 51, 745–752. 10.1136/jnnp.51.6.745 2841426 PMC1033142

[brb33097-bib-0017] Goetz, C. G. , Tilley, B. C. , Shaftman, S. R. , Stebbins, G. T. , Fahn, S. , Martinez‐Martin, P. , Poewe, W. , Sampaio, C. , Stern, M. B. , Dodel, R. , Dubois, B. , Holloway, R. , Jankovic, J. , Kulisevsky, J. , Lang, A. E. , Lees, A. , Leurgans, S. , Lewitt, P. A. , Nyenhuis, D. , … Lapelle, N. (2008). Movement Disorder Society‐sponsored revision of the Unified Parkinson's Disease Rating Scale (MDS‐UPDRS): Scale presentation and clinimetric testing results. Movement Disorders, 23(15), 2129–2170. 10.1002/mds.22340 19025984

[brb33097-bib-0018] Hillestad, E. M. R. , Van Der Meeren, A. , Nagaraja, B. H. , Bjørsvik, B. R. , Haleem, N. , Benitez‐Paez, A. , Sanz, Y. , Hausken, T. , Lied, G. A. , Lundervold, A. , & Berentsen, B. (2022). Gut bless you: The microbiota‐gut‐brain axis in irritable bowel syndrome. World Journal of Gastroenterology, 28(4), 412–431. 10.3748/wjg.v28.i4.412 35125827 PMC8790555

[brb33097-bib-0019] Hinkle, J. T. , Perepezko, K. , Mills, K. A. , Mari, Z. , Butala, A. , Dawson, T. M. , Pantelyat, A. , Rosenthal, L. S. , & Pontone, G. M. (2018). Dopamine transporter availability reflects gastrointestinal dysautonomia in early Parkinson disease. Parkinsonism & Related Disorders, 55, 8–14. 10.1016/j.parkreldis.2018.08.010 30146185 PMC6291234

[brb33097-bib-0020] Honkanen, E. A. , Noponen, T. , Hirvilammi, R. , Lindholm, K. , Parkkola, R. , Joutsa, J. , Varrone, A. , & Kaasinen, V. (2021). Sex correction improves the accuracy of clinical dopamine transporter imaging. Ejnmmi Research, 11(1), 82. 10.1186/s13550-021-00825-3 34424408 PMC8382816

[brb33097-bib-0021] Hubbard, C. S. , Becerra, L. , Heinz, N. , Ludwick, A. , Rasooly, T. , Yendiki, A. , Wu, R. , Schechter, N. L. , Nurko, S. , & Borsook, D. (2018). Microstructural white matter abnormalities in the dorsal cingulum of adolescents with IBS. eNeuro, 5(4), ENEURO.0354–ENEU17.2018. 10.1523/ENEURO.0354-17.2018 30109260 PMC6090517

[brb33097-bib-0022] Ito, K. , Morrish, P. K. , Rakshi, J. S. , Uema, T. , Ashburner, J. , Bailey, D. L. , Friston, K. J. , & Brooks, D. J. (1999). Statistical parametric mapping with 18F‐dopa PET shows bilaterally reduced striatal and nigral dopaminergic function in early Parkinson's disease. Journal of Neurology, Neurosurgery, and Psychiatry, 66(6), 754–758. 10.1136/jnnp.66.6.754 10329749 PMC1736402

[brb33097-bib-0023] Joling, M. , Vriend, C. , Raijmakers, P. G. H. M. , Van Der Zande, J. J. , Lemstra, A. W. , Berendse, H. W. , Booij, J. , & Van Den Heuvel, O. A. (2019). Striatal DAT and extrastriatal SERT binding in early‐stage Parkinson's disease and dementia with Lewy bodies, compared with healthy controls: An 123I‐FP‐CIT SPECT study. NeuroImage: Clinical, 22, 101755. 10.1016/j.nicl.2019.101755 30884365 PMC6424141

[brb33097-bib-0024] Jones, J. D. , Dominguez, B. , Bunch, J. , Uribe, C. , Valenzuela, Y. , & Jacobs, J. P. (2021). A bidirectional relationship between anxiety, depression and gastrointestinal symptoms in Parkinson's disease. Clinical Parkinsonism & Related Disorders, 5, 100104. 34430845 10.1016/j.prdoa.2021.100104 PMC8368023

[brb33097-bib-0025] Joutsa, J. , Martikainen, K. , Vahlberg, T. , Voon, V. , & Kaasinen, V. (2012). Impulse control disorders and depression in Finnish patients with Parkinson's disease. Parkinsonism & Related Disorders, 18(2), 155–160. 10.1016/j.parkreldis.2011.09.007 21983019

[brb33097-bib-0026] Kaasinen, V. , Kinos, M. , Joutsa, J. , Seppänen, M. , & Noponen, T. (2014). Differences in striatal dopamine transporter density between tremor dominant and non‐tremor Parkinson's disease. European Journal of Nuclear Medicine and Molecular Imaging, 41(10), 1931–1937. 10.1007/s00259-014-2796-5 24867256

[brb33097-bib-0027] Kano, M. , Muratsubaki, T. , Yagihashi, M. , Morishita, J. , Mugikura, S. , Dupont, P. , Takase, K. , Kanazawa, M. , Van Oudenhove, L. , & Fukudo, S. (2020). Insula activity to visceral stimulation and endocrine stress responses as associated with alexithymia in patients with irritable bowel syndrome. Psychosomatic Medicine, 82(1), 29–38. 10.1097/PSY.0000000000000729 31609924

[brb33097-bib-0028] Kim, H.‐S. , Cheon, S.‐M. , Seo, J.‐W. , Ryu, H.‐J. , Park, K.‐W. , & Kim, J. W. (2013). Nonmotor symptoms more closely related to Parkinson's disease: Comparison with normal elderly. Journal of the Neurological Sciences, 324(1‐2), 70–73. 10.1016/j.jns.2012.10.004 23102851

[brb33097-bib-0029] Kim, R. , & Jun, J.‐S. (2019). Association of autonomic symptoms with presynaptic striatal dopamine depletion in drug‐naive Parkinson's disease: An analysis of the PPMI data. Autonomic Neuroscience, 216, 59–62. 10.1016/j.autneu.2018.09.005 30236547

[brb33097-bib-0030] Krishnan, S. , Sarma, G. , Sarma, S. , & Kishore, A. (2011). Do nonmotor symptoms in Parkinson's disease differ from normal aging? Movement Disorders, 26(11), 2110–2113. 10.1002/mds.23826 21661056

[brb33097-bib-0031] Lai, S.‐W. , Liao, K.‐F. , Lin, C.‐L. , & Sung, F.‐C. (2014). Irritable bowel syndrome correlates with increased risk of Parkinson's disease in Taiwan. European Journal of Epidemiology, 29, 57–62. 10.1007/s10654-014-9878-3 24442494

[brb33097-bib-0032] Lee, J.‐Y. , Lao‐Kaim, N. P. , Pasquini, J. , Deuschl, G. , Pavese, N. , & Piccini, P. (2018). Pallidal dopaminergic denervation and rest tremor in early Parkinson's disease: PPMI cohort analysis. Parkinsonism & Related Disorders, 51, 101–104. 10.1016/j.parkreldis.2018.02.039 29503156

[brb33097-bib-0033] Liu, B. , Sjölander, A. , Pedersen, N. L. , Ludvigsson, J. F. , Chen, H. , Fang, F. , & Wirdefeldt, K. (2021). Irritable bowel syndrome and Parkinson's disease risk: Register‐based studies. npj Parkinson's Disease, 7(1), 5. 10.1038/s41531-020-00145-8 PMC7785733 33402695

[brb33097-bib-0034] Longstreth, G. F. , Thompson, W. G. , Chey, W. D. , Houghton, L. A. , Mearin, F. , & Spiller, R. C. (2006). Functional bowel disorders. Gastroenterology, 130(5), 1480–1491. 10.1053/j.gastro.2005.11.061 16678561

[brb33097-bib-0035] Lu, S. , Jiang, H.‐Y. , & Shi, Y.‐D. (2022). Association between irritable bowel syndrome and Parkinson's disease: A systematic review and meta‐analysis. Acta Neurologica Scandinavica, 145(4), 442–448. 10.1111/ane.13570 34908158

[brb33097-bib-0036] Ma, Y. , Dhawan, V. , Mentis, M. , Chaly, T. , Spetsieris, P. G. , & Eidelberg, D. (2002). Parametric mapping of [18F]FPCIT binding in early stage Parkinson's disease: A PET study. Synapse, 45(2), 125–133. 10.1002/syn.10090 12112405

[brb33097-bib-0037] Maass, A. , & Reichmann, H. (2013). Sleep and non‐motor symptoms in Parkinson's disease. Journal of Neural Transmission, 120(4), 565–569. 10.1007/s00702-013-0966-4 23338671 PMC3611039

[brb33097-bib-0038] Mertsalmi, T. H. , Aho, V. T. E. , Pereira, P. A. B. , Paulin, L. , Pekkonen, E. , Auvinen, P. , & Scheperjans, F. (2017). More than constipation ‐ Bowel symptoms in Parkinson's disease and their connection to gut microbiota. European Journal of Neurology, 24(11), 1375–1383. 10.1111/ene.13398 28891262

[brb33097-bib-0039] Mertsalmi, T. H. , But, A. , Pekkonen, E. , & Scheperjans, F. (2021). Irritable bowel syndrome and risk of Parkinson's disease in Finland: A nationwide registry‐based cohort study. Journal of Parkinson's Disease, 11(2), 641–651. 10.3233/JPD-202330 PMC8150653 33646176

[brb33097-bib-0040] Mishima, T. , Fukae, J. , Fujioka, S. , Inoue, K. , & Tsuboi, Y. (2017). The prevalence of constipation and irritable bowel syndrome in Parkinson's disease patients according to Rome III diagnostic criteria. Journal of Parkinson's Disease, 7(2), 353–357. 10.3233/JPD-160982 PMC5438471 28157108

[brb33097-bib-0041] Mulak, A. (2015). Brain‐gut‐microbiota axis in Parkinson's disease. World Journal of Gastroenterology, 21(37), 10609–10620. 10.3748/wjg.v21.i37.10609 26457021 PMC4588083

[brb33097-bib-0042] Murtomäki, K. , Mertsalmi, T. , Jaakkola, E. , Mäkinen, E. , Levo, R. , Nojonen, T. , Eklund, M. , Nuuttila, S. , Lindholm, K. , Pekkonen, E. , Joutsa, J. , Noponen, T. , Ihalainen, T. , Kaasinen, V. , & Scheperjans, F. (2022). Gastrointestinal symptoms and dopamine transporter asymmetry in early Parkinson's disease. Movement Disorders, 37(6), 1284–1289. 10.1002/mds.28986 35274368 PMC9314058

[brb33097-bib-0043] Nicastro, N. , Garibotto, V. , & Burkhard, P. R. (2020). Extrastriatal 123I‐FP‐CIT SPECT impairment in Parkinson's disease – The PPMI cohort. BMC Neurology [Electronic Resource], 20(1), 192. 10.1186/s12883-020-01777-2 32416724 PMC7229596

[brb33097-bib-0044] Pfeiffer, R. F. (2016). Non‐motor symptoms in Parkinson's disease. Parkinsonism & Related Disorders, 22(1), S119–S122. 10.1016/j.parkreldis.2015.09.004 26372623

[brb33097-bib-0045] Pfeiffer, R. F. (2018). Gastrointestinal dysfunction in Parkinson's disease. Current Treatment Options in Neurology, 20(12), 1–12. 10.1007/s11940-018-0539-9 30361783

[brb33097-bib-0046] Pilotto, A. , Schiano Di Cola, F. , Premi, E. , Grasso, R. , Turrone, R. , Gipponi, S. , Scalvini, A. , Cottini, E. , Paghera, B. , Garibotto, V. , Rizzetti, M. C. , Bonanni, L. , Borroni, B. , Morbelli, S. , Nobili, F. , Guerra, U. P. , Perani, D. , & Padovani, A. (2019). Extrastriatal dopaminergic and serotonergic pathways in Parkinson's disease and in dementia with Lewy bodies: A 123I‐FP‐CIT SPECT study. European Journal of Nuclear Medicine and Molecular Imaging, 46(8), 1642–1651. 10.1007/s00259-019-04324-5 31098748

[brb33097-bib-0047] Pirker, W. (2003). Correlation of dopamine transporter imaging with parkinsonian motor handicap: How close is it? Movement Disorders, 18(S7), S43–S51. 10.1002/mds.10579 14531046

[brb33097-bib-0048] Poirier, A. A. , Aubé, B. , Côté, M. , Morin, N. , Di Paolo, T. , & Soulet, D. (2016). Gastrointestinal dysfunctions in Parkinson's disease: Symptoms and treatments. Parkinson's Disease, 2016, 6762528. 10.1155/2016/6762528 PMC5168460 28050310

[brb33097-bib-0049] Tominaga, K. , Tsumoto, C. , Ataka, S. , Mizuno, K. , Takahashi, K. , Yamagami, H. , Tanigawa, T. , Kawabe, J. , Watanabe, T. , Fujiwara, Y. , Shiomi, S. , Watanabe, Y. , & Arakawa, T. (2015). Regional brain disorders of serotonin neurotransmission are associated with functional dyspepsia. Life Sciences, 137, 150–157. 10.1016/j.lfs.2015.07.023 26232557

[brb33097-bib-0050] Van Deursen, D. N. , Van Den Heuvel, O. A. , Booij, J. , Berendse, H. W. , & Vriend, C. (2020). Autonomic failure in Parkinson's disease is associated with striatal dopamine deficiencies. Journal of Neurology, 267(7), 1922–1930. 10.1007/s00415-020-09785-5 32162062 PMC7320937

[brb33097-bib-0051] Varrone, A. , Dickson, J. C. , Tossici‐Bolt, L. , Sera, T. , Asenbaum, S. , Booij, J. , Kapucu, O. L. , Kluge, A. , Knudsen, G. M. , Koulibaly, P. M. , Nobili, F. , Pagani, M. , Sabri, O. , Vander Borght, T. , Van Laere, K. , & Tatsch, K. (2013). European multicenter database of healthy controls for [^123^I]FP‐CIT SPECT (ENC‐DAT): Age‐related effects, gender differences and evaluation of different methods of analysis. European Journal of Nuclear Medicine and Molecular Imaging, 40(2), 213–227. 10.1007/s00259-012-2276-8 23160999

[brb33097-bib-0052] Visser, M. , Marinus, J. , Stiggelbout, A. M. , & Van Hilten, J. J. (2004). Assessment of autonomic dysfunction in Parkinson's disease: The SCOPA‐AUT. Movement Disorders, 19(11), 1306–1312. 10.1002/mds.20153 15390007

[brb33097-bib-0053] Zhang, M. , Yang, S. , Li, X.‐C. , Zhu, H.‐M. , Peng, D. , Li, B.‐Y. , Jia, T.‐X. , & Tian, C. (2021). Study on the characteristics of intestinal motility of constipation in patients with Parkinson's disease. World Journal of Gastroenterology, 27(11), 1055–1063. 10.3748/wjg.v27.i11.1055 33776372 PMC7985734

[brb33097-bib-0054] Zhang, X. , Svn, Z. , Liv, M. , Yang, Y. , Zeng, R. , Huang, Q. , & Sun, Q. (2021). Association between irritable bowel syndrome and risk of Parkinson's disease: A systematic review and meta‐analysis. Frontiers in Neurology, 12, 720958. 10.3389/fneur.2021.720958 34630293 PMC8492947

[brb33097-bib-0055] Ziebell, M. , Holm‐Hansen, S. , Thomsen, G. , Wagner, A. , Jensen, P. , Pinborg, L. H. , & Knudsen, G. M. (2010). Serotonin transporters in dopamine transporter imaging: A head‐to‐head comparison of dopamine transporter SPECT radioligands 123I‐FP‐CIT and 123I‐PE2I. Journal of Nuclear Medicine, 51(12), 1885–1891. 10.2967/jnumed.110.078337 21078806

