# Supplementary material for: Dopamine transporter binding in the brain is linked to irritable bowel syndrome in Parkinson's disease
Source: Brain Behav. 2023 May 30;13(7):e3097. doi: 10.1002/brb3.3097 (PMC10338780; doi:10.1002/brb3.3097)
Supplement: Supplementary file 1 — Supplementary Table S1 SBR values and Z‐scores in PD patients in putamen and nucleus caudatus. [file BRB3-13-e3097-s001.docx]

**Supplementary Table S1** SBR values and Z-scores in PD patients in putamen and nucleus caudatus

| Study Subject | Right  Caudate | | Left  Caudate | | Right Ant.  Putamen | | Left Ant.  Putamen | | Right Post. Putamen | | Left Post.  Putamen | |
| --- | --- | --- | --- | --- | --- | --- | --- | --- | --- | --- | --- | --- |
|  | SBR | Z-score | SBR | Z-score | SBR | Z-score | SBR | Z-score | SBR | Z-score | SBR | Z-score |
| H0018 | 2.16 | -2 | 1.77 | -2.9 | 1.79 | -2.38 | 1.59 | -2.92 | 1.41 | -2.91 | 1.23 | -3.02 |
| H0020 | 2.51 | -1.59 | 2.14 | -2.44 | 2.24 | -1.82 | 1.13 | -4.09 | 0.82 | -4.43 | 0.34 | -5.11 |
| H0022 | 2.38 | -2.16 | 2.56 | -1.95 | 1.57 | -3.33 | 2.27 | -2.19 | 0.68 | -5.07 | 0.98 | -4.16 |
| H0024 | 2.57 | -1.43 | 2.07 | -2.53 | 2.34 | -1.59 | 1.8 | -2.73 | 1.32 | -3.34 | 1 | -3.72 |
| H0028 | 2.4 | -1.91 | 2.01 | -2.8 | 2.49 | -1.48 | 1.53 | -3.42 | 1.29 | -3.57 | 0.92 | -4.05 |
| H0034 | 2.11 | -2.04 | 2.1 | -2.21 | 1.78 | -2.35 | 1.54 | -2.96 | 1.02 | -3.68 | 0.92 | -3.61 |
| H0047 | 2.79 | -1.24 | 3.43 | -0.17 | 1.96 | -2.5 | 2.72 | -1.18 | 1.13 | -3.98 | 1.83 | -2.29 |
| H0050 | 2.52 | -1.12 | 2.1 | -2.08 | 1.95 | -1.93 | 1.84 | -2.25 | 1.31 | -2.93 | 0.97 | -3.36 |
| H0051 | 1.82 | -2.64 | 1.78 | -2.88 | 1.35 | -3.18 | 0.86 | -4.34 | 1.24 | -3.27 | 1.1 | -3.29 |
| H0059 | 2.12 | -1.81 | 1.89 | -2.4 | 1.48 | -2.69 | 1.81 | -2.22 | 1.04 | -3.4 | 0.75 | -3.72 |
| H0062 | 2.48 | -1.36 | 2.13 | -2.18 | 2.37 | -1.31 | 1.85 | -2.39 | 1.67 | -2.35 | 1.07 | -3.32 |
| J0001 | 1.71 | -3.04 | 1.93 | -2.77 | 1.23 | -3.57 | 1.38 | -3.51 | 1.21 | -3.53 | 1.16 | -3.37 |
| J0002 | 2.51 | 1.25 | 2.54 | -1.33 | 1.84 | -2.22 | 1.82 | -2.39 | 1.38 | -2.9 | 1.45 | -2.5 |
| J0006 | 3.94 | 1.05 | 3.38 | -0.14 | 3.26 | -0.06 | 2.57 | -1.36 | 2.77 | -0.47 | 1.21 | -3.44 |
| J0007 | 2.94 | -1.36 | 3.86 | 0.27 | 2.48 | -1.93 | 2.87 | -1.3 | 0.98 | -4.73 | 1.93 | -2.51 |
| J0013 | 3.15 | 0.12 | 2.8 | -0.68 | 2.61 | -0.68 | 2.32 | -1.25 | 1.82 | -1.82 | 1.06 | -3.12 |
| J0023 | 3.27 | -0.48 | 3.64 | 0.09 | 2.16 | -2.3 | 2.88 | -1.03 | 1.07 | -4.28 | 1.56 | -3.01 |
| J0049 | 2.9 | -0.42 | 2.33 | -1.66 | 2.76 | -0.49 | 2 | -1.96 | 1.89 | -1.75 | 0.88 | -3.56 |
| J0050 | 2.22 | -2.04 | 2.5 | -1.66 | 1.82 | -2.48 | 1.74 | -2.79 | 0.71 | -4.54 | 0.81 | -4.05 |
| J0053 | 2.74 | -0.74 | 2.66 | -1.04 | 2.19 | -1.51 | 1.86 | -2.24 | 1.01 | -3.57 | 0.64 | -4.06 |
| J0055 | 3.24 | -0.22 | 3.08 | -0.67 | 2.44 | -1.49 | 1.92 | -2.57 | 1.4 | -3.26 | 1.07 | -3.67 |
| J0057 | 2.48 | -1.07 | 2.32 | -1.52 | 2.02 | -1.67 | 1.74 | -2.31 | 1.26 | -2.89 | 1.02 | -3.11 |
| J0063 | 1.49 | -3.34 | 1.58 | -3.32 | 1.19 | -3.52 | 1.12 | -3.89 | 0.62 | -4.6 | 0.91 | -3.72 |
| J0066 | 2.25 | -1.62 | 2.14 | -1.98 | 1.83 | -2.11 | 1.63 | -2.63 | 1.51 | -2.49 | 1.28 | -2.7 |

**Supplementary Table S1** SBR values and Z-scores in PD patients in putamen and nucleus caudatus (continued)

| Study Subject | Right  Caudate | | Left  Caudate | | Right Ant.  Putamen | | Left Ant.  Putamen | | Right Post. Putamen | | Left Post.  Putamen | |
| --- | --- | --- | --- | --- | --- | --- | --- | --- | --- | --- | --- | --- |
|  | SBR | Z-score | SBR | Z-score | SBR | Z-score | SBR | Z-score | SBR | Z-score | SBR | Z-score |
| J0068 | 3.02 | -0.46 | 2.96 | -0.72 | 2.97 | -0.37 | 2.07 | -2.1 | 2.21 | -1.38 | 0.68 | -4.26 |
| J0070 | 1.88 | -2.66 | 1.95 | -2.69 | 1.19 | -3.6 | 1.35 | -3.52 | 0.99 | -3.94 | 0.89 | -3.85 |
| J0076 | 2.69 | -0.96 | 3.05 | -0.41 | 1.87 | -2.22 | 2.26 | -1.58 | 0.87 | -4 | 0.97 | -3.52 |
| J0077 | 4.81 | 2.69 | 4.33 | 1.65 | 4.82 | 2.73 | 2.88 | -0.79 | 4.33 | 2.71 | 0.89 | -4.12 |
| J0087 | 2.23 | -1.66 | 2.64 | -1.01 | 2.15 | -1.54 | 2.35 | -1.23 | 1.41 | -2.69 | 1.5 | -2.25 |
| J0089 | 2.46 | -1.48 | 2.7 | -1.15 | 2.06 | -1.95 | 2.06 | -2.06 | 1.07 | -3.67 | 1.37 | -2.8 |
| J0092 | 2.24 | -2.01 | 1.64 | -3.3 | 1.93 | -2.28 | 1.43 | -3.39 | 1.43 | -3.05 | 1.04 | -3.58 |
| J0099 | 1.46 | -3.51 | 1.94 | -2.75 | 1.09 | -3.83 | 1.32 | -3.64 | 0.82 | -4.34 | 0.93 | -3.84 |
| J0102 | 2.25 | -2.35 | 3.62 | 0.12 | 1.7 | -3.05 | 2.98 | -0.76 | 1.28 | -3.76 | 2.5 | -1.01 |
| J0103 | 2.54 | -1.49 | 3.07 | -0.61 | 1.52 | -3.08 | 2.81 | -0.76 | 0.63 | -4.75 | 1.86 | -1.96 |
| J0104 | 1.94 | -2.4 | 1.97 | -2.48 | 1.11 | -3.59 | 1.54 | -2.98 | 0.95 | -3.84 | 1.05 | -3.36 |
| J0107 | 1.73 | -2.71 | 1.91 | -2.52 | 1.24 | -3.28 | 1.3 | -3.38 | 0.84 | -3.98 | 1.03 | -3.32 |
| J0109 | 2.51 | -1.75 | 2.69 | -1.55 | 2.06 | -2.29 | 1.85 | -2.85 | 0.78 | -4.68 | 0.77 | -4.41 |
| J0113 | 2.65 | -0.93 | 2.91 | -0.57 | 2.17 | -1.58 | 2.27 | -1.46 | 1.32 | -2.97 | 1.74 | -1.85 |
| J0114 | 2.78 | -0.92 | 2.37 | -1.86 | 2.47 | -1.26 | 2.11 | -2.01 | 1.84 | -2.14 | 1.39 | -2.81 |
| J0117 | 2.49 | -1.79 | 2.12 | -2.66 | 1.82 | -2.73 | 1.25 | -4.02 | 0.99 | -4.25 | 0.56 | -4.84 |
| T0057 | 2.16 | -2.1 | 2.51 | -1.57 | 1.58 | -2.87 | 2.01 | -2.21 | 0.79 | -4.32 | 0.67 | -4.28 |
| T0061 | 1.68 | -3.33 | 2.46 | -1.97 | 1.18 | -3.86 | 1.57 | -3.36 | 0.83 | -4.55 | 0.89 | -4.15 |
| T0062 | 3.01 | -0.08 | 3.03 | -0.18 | 2.23 | -1.31 | 2.07 | -1.67 | 1.18 | -3.08 | 1.08 | -3.02 |
| T0063 | 1.61 | -2.92 | 1.86 | -2.6 | 1.1 | -3.52 | 1.56 | -2.85 | 0.63 | -4.4 | 0.81 | -3.75 |
| T0064 | 1.71 | -2.64 | 1.92 | -2.39 | 1.03 | -3.55 | 1.14 | -3.57 | 0.51 | -4.54 | 0.71 | -3.85 |
| T0065 | 1.87 | -2.39 | 2.4 | -1.52 | 1.2 | -3.3 | 1.91 | -2.14 | 1.05 | -3.49 | 0.96 | -3.39 |
| T0074 | 2.12 | -2.49 | 2.84 | -1.26 | 1.32 | -3.62 | 1.95 | -2.63 | 0.9 | -4.42 | 0.87 | -4.21 |
| T0075 | 1.9 | -3.13 | 2.33 | -2.46 | 1.08 | -4.26 | 1.3 | -4.13 | 0.76 | -4.96 | 1.27 | -3.61 |
| T0077 | 1.43 | -3.1 | 1.24 | -3.62 | 0.89 | -3.74 | 0.84 | -4.09 | 0.67 | -4.15 | 0.6 | -3.99 |

**Supplementary Table S1** SBR values and Z-scores in PD patients in putamen and nucleus caudatus (continued)

| Study Subject | Right  Caudate | | Left  Caudate | | Right Ant.  Putamen | | Left Ant.  Putamen | | Right Post. Putamen | | Left Post.  Putamen | |
| --- | --- | --- | --- | --- | --- | --- | --- | --- | --- | --- | --- | --- |
|  | SBR | Z-score | SBR | Z-score | SBR | Z-score | SBR | Z-score | SBR | Z-score | SBR | Z-score |
| T0080 | 1.91 | -2.52 | 1.6 | -3.26 | 1.26 | -3.39 | 1.03 | -4.06 | 0.85 | -4.12 | 0.64 | -4.28 |
| T0089 | 1.79 | -2.72 | 1.55 | -3.33 | 1.72 | -2.52 | 1.1 | -3.88 | 1.02 | -3.74 | 0.71 | -4.1 |
| T0090 | 2.92 | -1.04 | 2.33 | -2.31 | 2.28 | -1.97 | 2.07 | -2.49 | 1.06 | -4.18 | 0.72 | -4.59 |
| T0098 | 1.26 | -3.48 | 1.75 | -2.7 | 0.87 | -3.84 | 1.07 | -3.71 | 0.59 | -4.37 | 0.81 | -3.65 |
| T0099 | 0.87 | -4.38 | 0.96 | -4.38 | 0.98 | -3.78 | 0.76 | -4.46 | 0.8 | -4.11 | 0.67 | -4.1 |
| T0101 | 1.26 | -3.51 | 1.35 | -3.48 | 0.98 | -3.64 | 0.68 | -4.47 | 0.62 | -4.32 | 0.38 | -4.53 |
| T0107 | 1.39 | -3.2 | 1.91 | -2.36 | 1.48 | -2.69 | 1.37 | -3.07 | 0.89 | -3.71 | 0.87 | -3.48 |
| T0123 | 1.98 | -2.38 | 1.94 | -2.61 | 1.8 | -2.42 | 1.48 | -3.18 | 0.79 | -4.26 | 0.46 | -4.64 |
| T0124 | 2.2 | -2.1 | 1.8 | -3.01 | 1.54 | -3 | 1.39 | -3.48 | 0.91 | -4.14 | 0.38 | -4.94 |
| T0125 | 2.56 | -1.83 | 2.36 | -2.36 | 2.12 | -2.36 | 1.42 | -3.85 | 1.32 | -3.75 | 0.58 | -4.97 |
| T0132 | 2.17 | -2.19 | 2.48 | -1.73 | 1.31 | -3.45 | 1.64 | -3.03 | 0.62 | -4.79 | 0.99 | -3.74 |
| T0147 | 1.97 | -2.3 | 1.94 | -2.51 | 1.87 | -2.19 | 0.97 | -4.07 | 1.55 | -2.57 | 0.58 | -4.29 |
| T0150 | 2.1 | -2.32 | 2.23 | -2.22 | 1.59 | -2.95 | 1.94 | -2.44 | 1.48 | -3 | 1.46 | -2.78 |
| T0163 | 1.23 | -3.5 | 1.21 | -3.71 | 0.74 | -4.02 | 1.12 | -3.56 | 0.93 | -3.61 | 0.65 | -3.92 |
| T0173 | 1.72 | -2.84 | 1.91 | -2.62 | 1.19 | -3.47 | 1.28 | -3.52 | 0.52 | -4.75 | 0.68 | -4.15 |
| T0177 | 1.94 | -2.37 | 2.91 | -0.66 | 1.37 | -3.1 | 2.25 | -1.59 | 0.93 | -3.85 | 1.65 | -2.11 |
| T0184 | 1.53 | -3.18 | 1.88 | -2.66 | 0.49 | -4.72 | 1.43 | -3.21 | 0.46 | -4.86 | 0.63 | -4.22 |
| T0188 | 1.72 | -2.8 | 2.06 | -2.29 | 1.52 | -2.83 | 1.91 | -2.25 | 1.25 | -3.2 | 1.18 | -3.08 |
| T0191 | 1.68 | -2.99 | 1.8 | -2.9 | 1.29 | -3.36 | 1.03 | -4.07 | 0.76 | -4.35 | 0.52 | -4.55 |
| T0192 | 1.75 | -2.69 | 1.79 | -2.76 | 1.69 | -2.47 | 1.75 | -2.5 | 1.38 | -2.88 | 1.34 | -2.69 |
| T0196 | 2.18 | -2.04 | 1.62 | -3.27 | 2.28 | -1.59 | 1.25 | -3.66 | 1.54 | -2.73 | 0.6 | -4.39 |
| T0197 | 1.52 | -3.09 | 0.78 | -4.66 | 1.24 | -3.24 | 0.61 | -4.68 | 1.19 | -3.22 | 0.5 | -4.36 |
| T0199 | 2.15 | -1.95 | 1.83 | -2.71 | 1.68 | -2.51 | 1.33 | -3.35 | 0.83 | -4.04 | 0.75 | -3.93 |

**Supplementary Table S1** SBR values and Z-scores in PD patients in putamen and nucleus caudatus (continued)

| Study Subject | Right  Caudate | | Left  Caudate | | Right Ant.  Putamen | | Left Ant.  Putamen | | Right Post. Putamen | | Left Post.  Putamen | |
| --- | --- | --- | --- | --- | --- | --- | --- | --- | --- | --- | --- | --- |
|  | SBR | Z-score | SBR | Z-score | SBR | Z-score | SBR | Z-score | SBR | Z-score | SBR | Z-score |
| T0201 | 1.98 | -2.01 | 1.86 | -2.39 | 1.25 | -3.06 | 1.16 | -3.43 | 0.79 | -3.84 | 0.52 | -4.12 |
| T0203 | 2.97 | -0.96 | 2.44 | -2.12 | 2.66 | -1.28 | 1.98 | -2.68 | 1.97 | -2.29 | 0.97 | -4.08 |
| T0211 | 1.44 | -3.16 | 1.4 | -3.38 | 0.76 | -4.05 | 1.26 | -3.33 | 0.54 | -4.49 | 0.93 | -3.4 |
| T0215 | 0.78 | -4.94 | 1.01 | -4.66 | 0.65 | -4.73 | 0.93 | -4.51 | 0.67 | -4.78 | 0.37 | -5.1 |
| T0219 | 1.43 | -3.12 | 1.82 | -2.52 | 1.11 | -3.35 | 1.75 | -2.32 | 0.68 | -4.14 | 1.21 | -2.78 |
| T0224 | 1.51 | -3.27 | 1.41 | -3.61 | 1.67 | -2.63 | 1.1 | -3.9 | 0.97 | -3.87 | 0.47 | -4.61 |
| T0225 | 1.37 | -3.35 | 1.72 | -2.84 | 1.06 | -3.55 | 1.15 | -3.61 | 0.6 | -4.42 | 0.39 | -4.57 |
| T0227 | 1.92 | -2.25 | 1.78 | -2.66 | 1.35 | -2.99 | 1.14 | -3.58 | 0.96 | -3.61 | 0.76 | -3.75 |
| T0233 | 2.93 | -0.65 | 2.78 | -1.09 | 1.92 | -2.28 | 1.45 | -3.32 | 1.08 | -3.75 | 0.89 | -3.85 |
| T0237 | 1.79 | -2.72 | 2.59 | -1.33 | 1.52 | -2.88 | 1.79 | -2.55 | 0.52 | -4.78 | 1.39 | -2.71 |
| T0238 | 2.24 | -2.24 | 2.6 | -1.69 | 1.77 | -2.8 | 1.93 | -2.66 | 1.1 | -4 | 1.14 | -3.63 |
| T0242 | 2.49 | -1.53 | 2.51 | -1.64 | 2.02 | -2.12 | 2.11 | -2.07 | 1.64 | -2.61 | 1.81 | -2.02 |
| T0246 | 1.61 | -3.09 | 1.25 | -3.92 | 1.13 | -3.61 | 1.24 | -3.64 | 0.41 | -5.03 | 0.59 | -4.36 |
| T0247 | 1.63 | -3.06 | 1.58 | -3.31 | 0.96 | -3.93 | 1.2 | -3.73 | 0.29 | -5.3 | 0.71 | -4.13 |
| T0250 | 2 | -2.64 | 2.16 | -2.49 | 1.44 | -3.35 | 1.56 | -3.32 | 0.73 | -4.7 | 1.08 | -3.7 |
| T0252 | 2.01 | -1.98 | 1.86 | -2.41 | 1.42 | -2.76 | 1.22 | -3.31 | 0.73 | -3.98 | 0.93 | -3.3 |
| T0259 | 1.5 | -3.13 | 1.57 | -3.14 | 1.28 | -3.18 | 1.09 | -3.76 | 0.65 | -4.36 | 0.46 | -4.46 |
| T0261 | 1.64 | -2.91 | 1.67 | -3.01 | 1.38 | -3.05 | 1.2 | -3.6 | 1 | -3.68 | 0.73 | -3.97 |

The Z-scores are based on healthy control sample. Abbreviations: SBR= spesific binding ratio, Z-score= Age-adjusted standard score; standard deviations from the mean
